# Febrile illness experience among Nigerian nomads

**DOI:** 10.1186/1475-9276-11-5

**Published:** 2012-01-31

**Authors:** Oladele B Akogun, Minnakur A Gundiri, Jacqueline A Badaki, Sani Y Njobdi, Adedoyin O Adesina, Olumide T Ogundahunsi

**Affiliations:** 1 Common Heritage Foundation, No.1 Bishop Street, Box 5124, Yola, Nigeria; 2 Federal University of Technology, Yola, Nigeria; 3 Adekunle Ajasin University, Akungba, Akoko, Nigeria; 4 Special Programme for Research and Training in Tropical Diseases, World Health Or-ganization, Geneva 27, Switzerland

## Abstract

**Background:**

An understanding of the febrile illness experience of Nigerian nomadic Fulani is necessary for developing an appropriate strategy for extending malaria intervention services to them. An exploratory study of their malaria illness experience was carried out in Northern Nigeria preparatory to promoting malaria intervention among them.

**Methods:**

Ethnographic tools including interviews, group discussions, informal conversations and living-in-camp observations were used for collecting information on local knowledge, perceived cause, severity and health seeking behaviour of nomadic Fulani in their dry season camps at the Gongola-Benue valley in Northeastern Nigeria.

**Results:**

Nomadic Fulani regarded *pabboje *(a type of "fever" that is distinct from other fevers because it "comes today, goes tomorrow, returns the next") as their commonest health problem. *Pabboje *is associated with early rains, ripening corn and brightly coloured flora. *Pabboje *is inherent in all nomadic Fulani for which treatment is therefore unnecessary despite its interference with performance of duty such as herding. Traditional medicines are used to reduce the severity, and rituals carried out to make it permanently inactive or to divert its recurrence. Although modern antimalaria may make the severity of subsequent *pabboje *episodes worse, nomads seek treatment in private health facilities against fevers that are persistent using antimalarial medicines. The consent of the household head was essential for a sick child to be treated outside the camp. The most important issues in health service utilization among nomads are the belief that fever is a Fulani illness that needs no cure until a particular period, preference for private medicine vendors and the avoidance of health facilities.

**Conclusions:**

Understanding nomadic Fulani beliefs about *pabboje *is useful for planning an acceptable community participatory fever management among them.

## Background

When compared to the urban populations, rural communities are poorly served by the health system, but in comparison with nomads, the gap between nomads and rural settled communities is even wider [1].

First, the formal health system appears ill-adapted for extending services to constantly mobile communities of nomads [2] and local authorities often disregard the existence of nomads with respect to health service delivery. For example, in Southwestern Nigeria, guinea worm case detection scouts "forgot" to include visits to nomad camps [3], and ivermectin distribution in the control of onchocerciasis, often marginalized nomadic Fulani settlements [4]. Located on the outskirt of settled communities, nomadic camps are often ignored to the extent that less than 3% of children below 2 years may benefit from full immunization service in some areas [5].

Although disproportionately more exposed to infectious diseases such as malaria, nomads remain isolated from the ongoing malaria management campaigns [6,7]. Accounting for more than 10% of Africa's overall disease burden, malaria is responsible for up to 20% of maternal deaths in health facilities in sub-Saharan Africa [8]. In Nigeria, malaria remains the most important cause of childhood morbidity and mortality. In 2000, malaria accounted for 63.4% of all reported diseases, with 50% of the population having at least one malaria episode leading to between 30 and 50% inpatient admissions and 50% outpatient visits to health facilities [9].

The typical Fulani household has a headman, his wives, children and dependents [10]. About 15 household units aggregate in an area to form a *wuro *(camp) and many *wuro *constitute a *gure*. Nomadic Fulani dependence on herbal remedy and private medical outlets in health seeking is well documented.

Despite current Government efforts to reduce the disease burden through distribution and promotion of insecticide treated nets (ITN) usage, simple diagnostic tools, behaviour change communication, appropriate chemotherapy, intermittent preventive treatment and community management of febrile illnesses, access to these services remain a challenge to the nomads. One of the major health issues in Africa is how beliefs about health and illness are used in health care delivery since the concept of illness is informed by cultural identity [11]. For example the Fulani express the sense of difference between them and other groups through the manner in which they acknowledge illness. Being Fulani means perseverance, strength of character, discipline and providing leadership over others. The lack of understanding of their cultural experiences with malaria is a hindrance to integrating them into the malaria control programme. Yet if not addressed, nomads will remain an epidemiologically significant group when the disease is eventually reduced to a level below public health importance among settled populations. A study on the malaria illness experiences of nomadic Fulani communities that camp in the valleys of rivers Benue and Gongola of Northeastern Nigeria during the dry season was conducted prior to development of an appropriate intervention programme.

## Methods

The study was carried out between November 2003 and April 2004. A mixed approach combining traditional biological techniques with ethnographic methods was used. Informal conversations, unstructured interviews, group discussion, and living-in-camp techniques were used for collecting information from nomads about their febrile illness experiences. These were then complimented with household and malaria prevalence surveys.

### Study population

The nomadic Fulani camp communities of interest were concentrated at the confluence of rivers Benue and Gongola in Northeastern Nigeria (Latitude 9°13'N and 9°46'N and Longitude 11°30'E and 12°10'E) spreading out over five Local Government Areas (LGAs). An extensive Savanna Sugar farm project irrigation scheme situated in the area provides abundant vegetation all year round. With an open vegetation of shrubs and herbaceous plants much favoured by animals, the area serves as a major dry season stop-post for nomads in their north-south migratory cycle. The camp is a settlement where the nomads make temporary shelter. The camp varies in size depending on the number of households inhabiting it. A camp often consists of several groups of concentric tents inhabited by a nomadic community. Several such communities may be located in a particular place within 5 kilometers of each other under a clan leader [10]. The clan is a subgroup within the larger Fulani tribe that claim descent from a common ancestor or share a perceived kinship. The clan often takes names of towns such as Jahun, Kiri, Bodewa to denote its origin or that of its founding ancestor. The sharing of the stipulated common ancestry is the symbol of the clan's unity. The migratory routes are clan based and the group passes through the symbol of the clan (such as Kiri for the Kiri Fulani clan) during a cycle of migration. It is not uncommon to find within the same clan, another set of groups that attempt to distinguish themselves from the other group members as a subclan based on an agnate of the same symbolic progenitor [10]. Members of the same clan usually camp in a particular area occupying sometimes about 3 kilometers radius, and share the same routes and movement timing.

Three main nomadic Fulani clans use the Benue-Gongola valley for their dry season stopover. At the peak of the dry seasons, forty-eight camps (estimated population of 12,000) dotted the entire area according to their clans. The Kiri undergo a migration within a radius of 30-100 kilometers and have a sedentary phase of old people, the Jahun migrate over a longer distance but use the valley on an annual basis while the Bororo (Bodyel clan) are cross-border nomads travelling over 800 km and only returning to the valley once in every three years. Most of the camps had moved in and out of the valley in the past 15 to 20 years. Members of the Kiri clan claim that as far as they could recall their great grandfathers had camped in the valley as well.

While the Kiri and the Jahun may spend up to 5 months in the valley the Bororo hardly spend 3 months before moving on. Camps of particular clans arrive and leave the valley at different times thus providing opportunity for collecting data from different members of the same clan throughout the dry season (November to May). A typical nomadic Fulani household unit consists of a set of concentric tents (built with reed, millet or corn stalks) in a semi-circle overlooking where animals are tethered. It is composed of a man, his sons, younger brothers, first cousins and other dependents. A household is a unit of economic activity headed by a *jom wuro*, or household head. The basic settlement of the Fulani is the *wuro *(camp) that is composed of 12-20 household units and headed by a *Jauro *or *bulama*. Each *wuro *is part of a cluster of several other *wuro *in an area belonging to the same clan. A collection of *wuro *is called the *gure *over which the eldest among brothers is the *Ardo *or clan leader. The *Ardo *wields the greatest influence in nomad leadership hierarchy. The *Ardo *has responsibility for coordinating movements, mediating disputes within the clan, negotiating with settled local community leaders or ordering a combat. The Savanna Sugar Irrigation farm project encouraged a set of camp sites with semi-permanent tents where in recent times the elderly nomadic Kiri Fulani and their children remain while the youth herd the animals within a radius of 50 and 100 kilometers. The elderly Kiri now combine mediatory and negotiation roles with trading in livestock at the local markets which may give rise to the sedentarization of the next generation. They do not cultivate crops at the moment. The Kiri keep goats and sheep in addition to cattle while the Jahun and Bororo keep only cattle.

An organization with previous experience working with nomads was consulted to learn how the nomads may be approached and their participation in the research negotiated. A local guide with experience carrying out veterinary services among the nomads and with whom the nomads had developed some trust was assigned to the team for the purpose of introducing and guiding the team through the social and cultural norms of the nomadic Fulani.

The *Sarkin sanu *or *Sarkin Fulani *(an informal position for a Fulani elder who serves as a liason for nomads at the local government office or at the traditional court of the local chief) was contacted and a list of *Ardo *was compiled and appointments made to visit. Every contact team visit had at least 3 research team members comprising culture specialist, facilitator, and a note-taker, one of whom is either a veterinary or health personnel. Diaries and field notes were used for documenting information and impressions.

### Team training

Members of the research team had previous experience in community interactive research and were fluent in Hausa and/or Fulfulde, the language of the Fulani. The research team received an orientation on Fulani culture. Since tape recording was not used at the onset to avoid suspicion and misunderstanding that are likely to occur during the first meeting, team members were trained on note taking techniques. In order to ensure quality, field notes were jointly reviewed by all the team members that were present during particular interaction.

### Contact and Informal conversations with clan leaders

During the first visit to the *Ardo*, the local guide introduced the team and initiated informal conversations about the health of their animals and their family. Conversations were then directed to febrile illnesses, access to insecticide treated nets and antimalaria medicines before the objective of the visit was introduced. In order to ascertain that the studied population would be around long enough to complete the study, inquiries were made about the clan's intended duration in their current location and willingness to participate in a study on febrile illness. The team returned to the *Ardo *at least three more times to offer specific follow up health and veterinary services while continuing informal conversations, gaining visibility and acceptance and developing useful contact for the subsequent stage. Services were limited to those within the professional competence of the Community Health Extension worker and the Veterinary Assistant in the team and include diarrheal and fever management and offer of referral support. The *Ardo *was encouraged to invite other elders of the clan (particularly the *Jauro*, the camp leaders) to the meetings. This provided additional opportunity for informal conversations with other elders of the clan and for documenting their knowledge and febrile illness experience. The conversations sought information on knowledge of local terminology, vocabularies and usages about febrile illnesses and malaria. Specific information on febrile illness recognition and classification, response to perceived cases of severe illness as well as how the health system served them was also sought. The local guide's role diminished with every subsequent visit and finally stopped when sufficient rapport had developed between the *Ardo *and the team. The provision of basic health and veterinary services during the study was very helpful in trust-building.

With the assistance of the *Ardo*, camps were selected from each of the community of camps. Clan representation and duration in the valley were important considerations in camp selection. The *Ardo *facilitated entry to the camps and introduced the team and the objective of the study to the *Jauro*.

### Formal interviews and discussions

Information from the informal contacts and conversations with clan elders was used for developing in-depth interview and group discussion guides. Group discussions were held with women (young, mothers, grandmothers), herders (young males) and children. In-depth interviews were held with camp leaders, local specialists (such as traditional healers). Specific questions on clinical signs, causality, meanings and perceived incidence rates were asked.

Much of the interaction was either at dawn or late in the evening in order to synchronize with their daily routine. The nomadic Fulani follow a very firm routine that commenced at about 4 hours (GMT+1) and ended at about 21 hours (GMT+1) when the animals had been tethered and the daily schedule completed. Informants were selected to represent relevant diversity in each community (age, gender, location and class). In order to avoid interview fatigue specific but few questions were asked about experience with febrile illness to the exclusion of other aspects that was discussed with other informants.

The data from the camps were used for developing household survey questionnaires that were administered to four members (male and female parent of under-five child, unmarried herders) of the household where formal interviews were not held. This allowed the collection of information from this category of people whose experiences would not otherwise be represented in the study, and to check for consistency and generalizability of information from the other sources. The data were then analyzed and the derived information used for developing an observation and informal conversation guides.

### Live-in experience

In order to understand the context within which information was obtained, three households with at least three under-five children and that are willing to host the team were selected from each clan for a 7-day live-in-camp experience. Typical family decision-making processes with respect to care-giving, health and illness behavior such as gender roles, extended family influence and age were documented in the process. Attention was given to family care, recognition of febrile illnesses, home remedies (orthodox or traditional), health service usage, nursing care and recuperation. The 3-member team comprised a health and veterinary assistant led by a facilitator (teams A, B, C). The team members shared the same tent built by the women and interacted within limits of the culture. Each member of the team selected a particular skill to acquire during the live-in experience. For example the woman member spent much of the time with the eldest woman in the camp but interacted with other women participating in their everyday tasks (learning to milk the cow, prepare yoghurt or millet meal). Similarly, a male member of the team spent much of the time with the herders and participated in herding and tendering the animals. The third member shared the time with the elders of the clan and where decorum permitted, accompanied the Ardo in some of the missions to the local community and markets, and to resolve misdemeanors at the local court. These offered opportunity for obtaining information on their migration and fever management practices. The arrangement also allowed the researchers to be as unobtrusive as much as possible. Notes were made by each team member at intervals. Every evening the team shared information and compared observations. The evening discussions also provided opportunity for identifying issues that required further understanding.

### Malaria prevalence survey

A subteam (D) carried out a malaria prevalence survey among under-five year old camp members using a rapid diagnostic test kit, ParaHit *f *developed by Program for Appropriate Technology (PATH) and manufactured by ICT Diagnostics, South Africa. ParaHit f is an immunodiagnostic test that provides in vitro qualitative test for the detection of *P. falciparum *(Pf)-specific Histidine Rich Protein II [12,13]. Rapid diagnostic test kits are useful for the prompt malaria diagnosis in community surveys [14]. The current study is preliminary to an investigation on malaria management among children under the age of five years since they are especially vulnerable.

The quantitative approach was used to assess malaria prevalence in children and cultural epidemiological indicators: frequency of self-reported experience of various diseases in order to estimate the perception of disease burden and agreement on causation. Qualitative approach assessed the domains of knowledge, causation, severity, perceived burden and pattern of treatment seeking response to illness. Quantitative data were entered into EPI INFO (version 3.3.2) and analyzed with IBM SPSS Statistics version 18 while qualitative data were processed with Atlas Ti.

## Results

Shrubs and herbaceous plants characterize the Benue river valley attracting nomads to the limited grazing areas particularly during the dry season. Although 28 camps were counted during the study, the team was informed that there were probably 160 camps at different locations with a total estimated population of about 10,000 nomads at the time. The initial realization was that the term *Bororo*, which non-Fulani people erroneously use to describe all Fulani nomads only apply to a particular clan. Those who did not belong to the clan felt offended when erroneously referred to as *Bororo*. The research team talked to more than 72 nomads during the qualitative data collection while 97 respondents participated in the survey. More males participated in the survey (60.8%) than females (39.2%). The malaria survey involved 691 under-five children.

The test kit detected only *Plasmodium falciparum *parasite antigen in blood. Infection was similar in both male (37.0%) and female (36.6%) but not between age-groups (Table 1).

**Table 1:** Prevalence of malaria infection among under-5 children

	No. Examined	% Infected	Significance
**Age (years)**			
≤ 1	237	27.4	P < 0.05, Chisqr: 18.6, Significant
2	151	45.5	
3	131	34.1	
4	172	45.1	
			
**Sex**			
Male	349	37.0	P > 0.05, Chisqr: 0.013, Not Significant
Female	342	36.6	

Infection was lowest among children that were less than 12 months but highest among two and four-year olds. Clan was not an important factor in infection with malaria parasite

### Clans, camps and lifestyle

Altogether 72 individuals participated in either informal conversations, group discussions or in the key informant interviews. The women and children sleep inside the tents while the men sleep outside, unprotected from mosquito bites. The Bororo children (5-12 years) sleep among the animals to "prevent mosquito bites". Irrespective of the clan camp members' duties and interactions are segregated by age and sex (elderly, married male, unmarried male, married female, unmarried female) and in-laws could not socially mingle. Responsibilities and duties are well defined. Women take care of food, children, and the tents while the young herd the animals. The elders serve as liaison to other clans and to the local community population. They gather news about pasture, potential conflict and patterns of local politics that may affect them. Among the Bororo and the Kiri, herders would spend about one hour studying in Arabic at the end of the day's work.

Among the Jahun and the Kiri, young girls (7-11 years) participate in herding. The elders and women patronize the nearest markets while the young tend the animals. Only the very young and infirm are left in the camp during the day. Information gathering and communication is very critical to the nomadic lifestyle. The *Ardo *spends a significant proportion of the time in the court of the sedentarized local community chief or in the market scooping information that could be useful for the safety of camp members and animals. A camp could relocate within a few minutes of a decision to do so. The Ardo is responsible for the decision to relocate. The use of mobile handheld phones is very common among the nomads for enhancing communication between the herders and the Ardo. The team (A, B) witnessed the relocation of five camps comprising thousands of heads of cattle to a position of about 25 kilometers upstream within an hour to avoid a local crisis which turned out to be a ruse.

The Bororo youth (both male and female) plait and adorn their hair and clothes with brightly coloured ornaments. A radio (with which they are linked to the outside world) is an indispensable part of dressing as they herd animals in the fields. The Jahun are witty, inquisitive but extremely wary of outsiders.

Camp live-in observations posed some challenge as words went round that the team was interested in health. At the onset many ailments were brought to the team. However the splitting of the team helped reduce the number as it became known that they would only meet the health personnel member of the team in the night or early in the morning. The provision of basic health care could pose a major obstacle to obtaining information on health among nomads using the live-in approach. However this is compensated by a rapid built-up of trust and rapport and enrichment of the quality of information collected during lay conversations and discussions while in the camp.

Very few childhood fevers were managed in the camp during the live-in period. On two occasions the household head brought antibiotics purchased from the local market for the treatment of diarrhea in a 7-year old. Although we were told that this was a common practice, the particular action was as a result of our presence otherwise we noted that attempts were made to exhaust other home remedies before purchasing modern medicines from local medicine vendors. The team witnessed four cases of childhood fever management while the health team member was with the herders and could not return to camp for the night. The observations were corroborated with information from conversations and discussions. The household head on receiving information about a member of the household being ill will assess the severity. The opinions of other men and in some cases suggestions of the old women were taken to account. Often the household head will not make known his decision but would leave the camp only to return later in the day with medicines for the treatment of the ill person. In exceptional cases the individual may be taken to a health facility but the team did not witness this. The team had an opportunity to observe a *boka *(medicine man) manage a non-malaria ailment. The women seemed to have a greater influence on the decision of the household head than acknowledged. They often influence the household head by the manner in which the ailment is described. In order to influence the household head's decision they often give examples of those with similar illness in some other place and how or where it was managed. Although the decision-maker often ignored the women the eventual action seemed to have the influence of the older women who served as nurse and administer medicines to children and other women.

It was much easier to observe the herders than activities in the camp. The presence of a guest had very little effect on their activities. The herders easily developed rapport and were eager to share skills, information and knowledge once in the field. In the camp they appeared shy and withdrawn in the presence of the older men. The rapport with team member in the field and the comradeship was much stronger than what the female team member witnessed among the women. Except the illness was so severe as to prevent herding the herders were often left to resolve any health issues they may encounter while herding. They freely purchased whatever medicines they wanted and used them at will even in the absence of any ailment. On many occasions, the herders would purchase vials of injectable chloroquine or antibiotics, break it with the teeth and swallow the content. It was believed to be more effective than pills in the absence of an injection. There was very little regard to expiration or distinguishing between antibiotics and antimalaria and would sometimes take multiple medicines at the same time. The herders would chew herbs and roots of various plants as well as modern medicines for different reasons: to keep warm, prevent or halt an emergent fever, or colds.

### Cultural epidemiology of fever

Nomads perceived *Pabboje*, a type of fever that recurs on alternate days as the most common health problem with 87.6% reporting that they had within the past six months (Table 2).

**Table 2:** Frequency of diseases in the camps

Self - reported diseases	Number interviewed	Frequency (%)^1^
*Pabboje*	374	87.6
Ringworms/Skin problems	84	19.7
Diarrhoea and abdominal pain	74	17.3
Back and joints pain	56	13.1
Dysentery	38	8.9
Worms	27	6.3
Cough	16	3.7
Others	3	0.7
Number of respondents	427	100.0

^1 ^Proportion reporting they had the condition

They acknowledged other ailments of concern to nomads as ringworms (19.7%) and diarrhoea (17.3%), Cough (*dambi*), and foot cracking (*pe-i*).

They described symptoms of *pabboje *as hotness of the body, fatigue, headache, shivering, pains in the joints, diarrhoea, vomiting and loss of appetite. *Pabboje *is nascent in the Fulani but can be triggered into active form when one drinks fresh milk during early rains, eats or perceives the aroma of fresh maize being roasted or cooked, or observes brightly coloured agents (red or yellow) such as maize flowers (Table 3).

**Table 3:** Factors that trigger *pabboje *(N = 97)^1^

Factors/agents	Frequency (%)
Fresh milk during the early rains	71.1
Fresh maize during the early rains	48.5
Roasted maize in early rains	36.1
Sight or aroma of maize	11.3
Consumption of maize	6.2
Greeting a person with *pabboje*	4.1
Others (e.g. bright colours, flowers)	20.6

^1^multiple responses allowed

They noted that however, the frequency and severity differs between individuals. While some have a single episode annually others have up to four, at onset or end of the rain and others at both seasons.

The symptoms of *pabboje *include weakness of the body, *naadaago *(stretching of the body), feeling cold and desire to sit in the hot sun, lack of appetite. Other diseases may exhibit similar symptoms but *pabboje *is unique because it gives intermittent respite to enable one carry out one's duties.

*Once it did not go away on the next day, we begin to suspect other types of djonte which could be caused by witches or evil spirits but we must wait for a week to be certain it is not pabboje *---informal conversation, Ardo, Bororo

A group of Fulani women articulated the generally held view

*Pabboje is natural with every Fulani and is not caused by anything. It is harmless but when one eats, smells or sees something pabboje does not like it wakes up and gives a little trouble which disturbs ones work*.---Focus group discussion, married women.

*My pabboje becomes unhappy when I drink milk during the early rains. Many people don't have pabboje during the dry season although they drink milk at that time. It can only be because the milk we drink in the rainy season is different. Cattle graze on fresh grass in the rainy season the milk from such cattle may be the one that wakes up the sleeping pabboje in the Fulani. ---*Indepth interview, healer and Jauro, Bororo

### Perceived severity and Patterns of Distress

Most Fulani nomads had experienced at least two annual episodes of *pabboje *but they do not associate it with death. Although *pabboje *was merely a condition for being a Fulani, and not an illness, nevertheless it was mentioned as cause of depression since it interfered with performance of normal tasks and with carrying out one's responsibilities in the camp.

*Pabboje starts with general body weakness and a desire to sit in the hot sun. One will then have naadaago (a kind of body stretching that happens when one has just woken up), the body will be hot, intense headache but one will feel cold and begin to shiver. Some people will vomit.----*Key informant Interview, Young male herder, Jahun

*Pabboje behaves differently in different people. May be this is because of what wakes it up. I don't know. In some people, pabboje will be so mild that it does not interfere with herding while it will be very severe in others.--*Informal conversation, herder, Bororo

*When I used to go herding, my pabboje would not come in the morning but will allow me take the animals far afield then it will start when the sun is on top of the head, at about noon. I would then have to return home. I know the following day it would not come so I would go herding the animals. But on the third day, I would wait in the tent till noon for it to come and surely it would come. That is how I used to manage the three and sometimes four episodes that I used to have every year*.- Informal conversation, Ardo, Kiri

The distinguishing symptom of pabboje is that act of coming on days one, three, five before dis-appearing. Djonte is like pabboje that does not go away but that remains persistent

*Djonte is very dangerous. It will not allow you to herd the animals at all*.

---Informal conversation, Male herder, Kiri

The nomads would ignore fever until the third recrudescence. However if symptoms persisted beyond the 7th day, it was classified as *djonte *(fever) due to other causes and not *pabboje*. Nomads are relieved when *djonte *is due to *pabboje *and perceive other types of fever with much anxiety.

However, its interference with duties has made it necessary to curtail its recurrence or to reduce its severity to a level that normal activities such as herding could continue. Some traditional method of preventing the recurrence of *pabboje *is, at dawn after the first recurrence to take a handful of *lebol *(butter from cow milk), venture far into the bush, and place the *lebol *between the leaf of *barkeje *(a shrub with broad leaves), fold the leaf and then let go of it immediately. One should neither look back nor ever return to the particular spot. The next person that passes would take the illness from the victim and thus hinder subsequent episodes from emerging. Another approach is to cut an un-ripe pawpaw fruit into four equal parts and soak them in milk that is been prepared for *pendiidam *(yoghurt) and allow to ferment. The pawpaw would then be removed and the yoghurt served to the patient to drink. This will reduce the severity of *pabboje*. Cotton leaves may also be boiled and drunk in order to reduce the severity of *pabboje*. Yet, another antidote is to take a handful of guinea corn with the left hand and bury it under *dingaali *(a woody plant with broad bitter leaves) on the second day of *pabboje*. This action will bury the *pabboje *within, and prevent it from "waking up" on the third day. In some cases pabboje may remain buried forever unable to wake up even when triggered.

*Until recently, I did not know any modern treatment for pabboje. I would wait until the episodes of my pabboje has come and passed*--- Informal conversation, Ardo Kiri

Some women keep antimalaria purchased from local vendors at home for treating pabboje in children.

*At the store we just ask for medicine for ciwonfulani (*Fulani problem). *We give two tablets. If the child is too young, we give syrup. The child vomits and then becomes well again. Pabboje starts in the night or early in the morning--*Focus group discussion, Young mothers, Kiri

The elderly believe that the treating *pabboje *had "made it more severe" and many would rather not use any modern medicines in cases of *pabboje*.

*Modern drugs, like quinine for treating pabboje disappeared then there was nivaquine and later chloroquine injection which was very effective with some people. However, many of us do not treat pabboje because modern drugs will only make pabboje go away for a short time only to return with more virulence. The only drug I did not hear people complain about is the one your group brings to us*.----Indepth interview, Ardo, Jahun

*Pabboje *is distinguished from *djonte *because the former recurs while the latter does not. Whenever someone has *djonte *it will be assumed to be *pabboje *except when proven otherwise. The main proof is for it not to go away and comeback again. There are other types of *djonte *which the nomads consider very dreadful since they interfere severely with normal activities. If it is not *pabboje*, then it is caused by evil spirits or witches and should be treated by placing leaves of baobab on red hot charcoal and inhaling the smoke.

One such *djonte *is d*jonte kippindirde *considered the most precarious of the *djonte *and can only be removed by traditional healers since it is caused by *hendu *(evil spirits) or *mutte'en or karama'en *(witches).

*Buudi *or *Buule *(plural) may also cause *djonte*. Fever due to *buudi *is milder than *pabboje*. Although the victim feels weak and has hot body there will neither be shivering, or feeling cold. Although *buudi *may subside and escalate; it does not follow any regular pattern like *pabboje. Buudi *can be felt when the inflamed part is touched.

Besides *djonte, pabboje*, other diseases that nomads regard as intrinsic waiting to be externally triggered are *Doiru *(tuberculosis), *peewol (*rheumatism), and *sadaure *(leprosy or dermatitis).

Nomads take measures to protect themselves from mosquitoes because they are a nuisance, suck blood, and may cause itching at the point of bite. Insecticide treated nets, clothing (especially when asleep), herbs burned to create smoke and plant repellents are used to prevent man-mosquito contact. Herders hang certain plants around the body while in the field and place some at different parts of the tent to repel mosquitoes. Among the Bororo, children sleep among the large animals such as cattle to prevent mosquito bite. An 8-year old nomadic Fulani child spoke for his colleagues

*We sleep among the cows so that the mosquitoes do not see us to bite. The cattle are big and the mosquitoes bite them instead*.

### Decision-making and health service utilization

Since the live-in exercise did not yield much information from observed practices, the team depended on conversations and the survey results. Although most nomadic Fulani (79.4%, N = 97) knew the location of the nearest health facilities, only a few (5.8%) visited or knew someone who had visited any in the past six months. Self-treatment with a variety of antimalaria drugs and antibiotics is very common particularly among the young male (herders). We observed them purchase antibiotics and vials of injectable medicines displayed on a mat laid on the market floor, break the vial open with the teeth and swallow the content.

The mother of a febrile child would inform the father whose responsibility it was to decide the course of action. When an adult was severely ill and unable to communicate, the most elderly male would decide the course of action to be taken. When illness is severe enough as to be life threatening one in ten women would take a child to the health facility without the consent of the father. Male children informed their fathers when they fell ill while female children notified mothers. If the head of a family was not available, the brother or other person to whom the household was entrusted would take decisions in his absence. However, no action would be taken until the 7th day when the type of *djonte *is determined. If fever persisted without any recurrent pattern it would be assumed to be severe and treated as such. Once a decision was made the father would allocate resources (money, donkey and advice) to enable the mother implement it. It is in rare cases that a father would take the child to the health facility.

In recent times, nomadic Fulani patronise private health facilities but hardly public ones. The reasons were similar irrespective of clans or camp. Payment condition (49.5%), distance from camp (42.3%) and the politeness of the health care provider are the most important considerations when making a decision to patronise a health facility. Nomads spend as much as $8 in the purchase of antimalaria medicines most often (of unknown and doubtful origin) from itinerant vendors at local markets (Table 4).

**Table 4:** Reasons for preferring one source of intervention to the other (N = 97)^1^

Reasons	Frequency (%)
Payment condition is flexible and can be negotiated	49.5
Distance: the place is not far from campsite	42.3
Time spent in the facility is short and convenient	38.1
Service is good and relief is obtained	30.9
Staff of facility are friendly and respect the Fulani	22.7
Type of drug and mode of dispensing can be negotiated	22. 7
Services and supplies are always available	20.6
Others	2.0

^1 ^multiple responses allowed, vertical comparison only

Nomads are very much concerned about their inability to benefit from regular health service and expressed willingness to participate in the distribution of insecticide treated nets (ITN) and in the administration of antimalaria for the management of fevers in the camps. However they expressed concern about time and duration of training as well as how participation would affect their nomadic life. If required they would be keen to contribute to the purchase of drugs, to support the camp own resource person (CORP) during training and to compensate for time if the acquired skills would be used solely for the benefits of fellow camp members.

## Discussion

That majority of the camps had moved in and out of the same area for more than 15 years confirm the predictability of migratory patterns of nomadic communities. Their highly developed communication skills and respect for hierarchy are advantages that health authorities could take for developing appropriate intervention programme that nomads would accept.

The recorded prevalence of malaria in the survey is not surprising. A previous study in Northeastern Nigeria reported up to 58.7% prevalence of *Plasmodium falciparum *[15]. Malaria transmission in northeastern Nigeria is seasonal and reaches the peak at the onset (May/June) and end (September/October) of rainy season when anopheline mosquito breeding sites are abundant. Although the breeding sites dry up in most parts of Northeastern Nigeria during the intensely hot and dry months (February/April), focal transmission abound in isolated sites such as water holes and perennial river beds. Nomads are exposed to this focal but intense transmission when they camp near water holes in consideration of the need of their animals. Unlike most sedentary communities, nomadic populations are therefore exposed to malaria transmission throughout the year and thus deserve special attention by control programmes. In Mali, disproportionately low prevalence of malaria among Fulani tribe when compared with other tribes had been associated with an unidentified genetic factor [16]. Such association is difficult to infer in this study.

The nomadic Fulani have a highly well developed theory of causation of febrile illnesses and are able to distinguish between different types of fevers. The perception of *pabboje *as different from other types of *djonte *but personal to the Fulani nomads is probably, as Imperato [17] explained, a means of coping with the disease. Whereas *pabboje *provided respite for them to meet their obligations and duties to their family and animals (*pulaaku*) other types of *djonte *do not and are therefore dreaded. This is perhaps the reason for regarding *pabboje *as a problem of the nomadic Fulani population. The intermittent distress due to *pabboje *still provides some respite for performing one's duty as Fulani. The fact that it is regarded as nascent, requiring a trigger factor to make it come alive probably explains the focus of traditional intervention which is to negotiate the return of *pabboje *to its dormant status without recurring or to reduce its severity but not completely clearing it.

The gradual acceptance of treatment of *pabboje *indicates a change in awareness that malaria control intervention programmes need for developing a control programme for the nomads. The market which serves as a key meeting point for collection and dissemination of information among nomads could be exploited by the health authority for interactive communication.

Nomads will most likely continue to patronize the private health facility abandoning the public health facility to sedentary community populations as observed in another study in Nigeria [18]. It is unlikely that communities could be convinced to accept the sharing of limited consumable health resources with temporary guests whose population and health needs may often overwhelm that of the local population. In Nigeria allocation of resources and services are carefully computed and shared between spheres of governance at the federal, state, local government area and community according to set criteria which takes into consideration the census of the community. The nomads are not often included at the planning stage. Although communication technology-driven empowerment combined with simple rapid diagnostic tools and creative packaging of medicines may increase access to fever management services, it is yet to attract the attention of potential investors [19] The most plausible strategy is an intervention that is nomadic community-managed, and that exerts minimal pressure on pre-allocated resource of the sedentary community.

The community directed intervention (CDI) approach that has been made popular in the control of onchocerciasis in Africa is probably the most viable option for increasing access of the nomads to malaria intervention services [20,21]. The approach requires that the health authorities and partners negotiate the means by which the community will participate in intervention to ensure ownership of intervention process and outcome. First the nomads' migratory movement and settlement patterns within a health district should be identified, described and a plan developed for approaching and negotiating their participation in malaria control. The cultural epidemiological description of the disease is also useful for planning intervention strategies particularly the development of information, education and communication strategies.

## Conclusions

Despite the view expressed by some nomads that the use of Western medicine might have worsen the disease, majority attest to the relief offered by Western medicine and accept it if it is made accessible to them. The community directed intervention strategy is the basis for increasing that accessibility. The challenge before the health system is to apply the community directed intervention approach that has worked well with settled communities to the nomadic lifestyle.

## Competing interests

The authors declare that they have no competing interests.

## Authors' contributions

OBA conceived, designed and coordinated the study; MAG and JAB reviewed the protocols and improved the intellectual content while SN and AOA coordinated prevalence survey, analyzed and interpreted the data, OTO contributed to the design and reviewed the final manuscript for intellectual quality. The protocol had the ethical approval of the National Health Research Ethics board of Nigeria and the ethics review committee of the World Health Organization. All authors read and approved the final manuscript.
